# 实时定量PCR 2^-ΔΔCt^法检测非小细胞肺癌*HER2*基因的过表达

**DOI:** 10.3779/j.issn.1009-3419.2011.12.07

**Published:** 2011-12-20

**Authors:** 水东 奉, 红专 谭, 宏艳 凌, 秀琴 袁

**Affiliations:** 1 421001 衡阳, 南华大学公共卫生学院社会医学与卫生事业管理教研室 Department of Social Medicine and Health Services Management, School of Public Health, University of South China, Hengyang 421001, China; 2 410087 长沙, 中南大学公共卫生学院流行病与卫生统计学教研室 Department of Epidemiology and Health Statistics, School of Public Health, Central South University, Changsha 410087, China; 3 421001 衡阳, 南华大学医学院生理学教研室 Department of Physiology, School of Medicine, University of South China, Hengyang 421001, China

**Keywords:** 实时定量PCR, *HER2*基因, 肺肿瘤, 表达, Real-time quantitative PCR, *HER2* gene, Lung neoplasms, Expression

## Abstract

**背景与目的:**

正确评价*HER2*基因表达的状况对于肿瘤的施治具有重要的指导意义。以往的评价大多基于免疫组织化学法, 但结果变异性大。本研究旨在探讨实时定量PCR 2^-ΔΔCt^法检测非小细胞肺癌(non-small cell lung cancer, NSCLC)*HER2*基因表达的可行性。

**方法:**

采用实时定量PCR 2^-ΔΔCt^法同时检测212例肺癌组织和与其匹配的非肿瘤组织*HER2*基因的表达, 评价过表达水平。

**结果:**

肺癌组织中*HER2*基因的表达水平高于非肿瘤组织(T=67, *P* < 0.05), *HER2*基因的过表达率为34.0%。

**结论:**

实时定量PCR 2^-ΔΔCt^法用于检测NSCLC *HER2*基因的表达是可行的。

## Abstract (en)

**Background and objective:**

The evaluation of the expression level of the *HER2* gene for the diagnosis and treatment of tumors is usually conducted using immunohistochemical techniques.The aim of the current study is to explore the feasibility of real-time quantitative PCR and the 2[-Delta Delta C(T)] method in detecting the level of *HER2* gene overexpression in non-small cell lung cancer (NSCLC).

**Methods:**

Real-time quantitative PCR and the 2[-Delta Delta C(T)] method were used to detect the level of *HER2* gene overexpression in 212 lung cancer and matched non-tumor tissue specimens.

**Results:**

The expression level of *HER2* gene in lung cancer tissue was higher than that in the matched non-tumor tissue, with an overexpression rate of 34%.

**Conclusion:**

Real-time quantitative PCR and the 2[-Delta Delta C(T)] method can be used to detect the level of *HER2* gene overexpression in NSCLC.

*HER2*基因过表达能增强肿瘤的生长、恶性型变及侵袭力, 且与较差的临床预后和化学药物耐受有关^[1]^, 因此, 正确评价*HER2*基因表达的状况对于肿瘤的治疗、干预、预后估计及抗肿瘤药物的筛选等具有重要的指导意义。研究^[2, 3]^发现非小细胞肺癌(non-small cell lung cancer, NSCLC)*HER2*基因突变的发生率很低, 但*HER2*基因的过表达却较常见, 因此常通过检测*HER2*基因的过表达来探讨*HER2*基因在肺癌发生、发展中的作用。目前各研究报道的*HER2*基因过表达率不一致, 这主要与检测方法的选择以及方法的敏感性有关。*HER2*基因表达的状况大多采用免疫组织化学法(immunohistochemistry, IHC)来进行评价^[4-6]^, 但该方法的变异性较大, 操作过程中容易受各种主、客观因素的影响, 如判断标准、抗体的敏感性以及不同的实验条件等都可能产生不同的阳性率。因此, 采用更为客观的方法评价*HER2*基因在肺癌中的表达很有必要。

近年来出现的实时定量PCR(real-time quantitative PCR, RT-Q-PCR)技术实现了PCR从定性到定量的飞跃, 它以特异性强、灵敏度高、重复性好、定量准确、速度快、全封闭反应等优点成为了分子生物学研究的一个重要工具。因此本研究尝试采用RT-Q-PCR评价NSCLC *HER2*基因的表达水平, 以期为肺癌临床干预策略的制定提供依据。

## 1 对象与方法

### 1.1 对象的来源

选择2006年6月-2007年6月在湖南省肿瘤医院、中南大学湘雅医院和中南大学湘雅二院三所大型医院收治住院并通过病理组织学确诊为NSCLC的所有患者作为研究对象, 收集其肺癌组织和与其匹配的非肿瘤组织标本各212例, 于-80 ℃冷冻保存。其中包括男性172例, 女性40例。病理组织学类型为:腺癌46例, 鳞癌126例, 腺鳞癌37例, 肺泡细胞癌3例。TNM分期:Ⅰ期66例, Ⅱ期50例, Ⅲ期70例, Ⅳ期26例。

### 1.2 实验方法

#### 1.2.1 主要仪器

包括:SDS-PAGE微型电泳仪及电泳槽(BioRad公司), 普通梯度PCR仪(BioRad公司), 低温高速离心机(Eppendorf公司), 紫外分光光度计(Beckman公司), 凝胶成像分析系统(Alpha公司), 瓷研钵(民生堂大药房), icycler iQ荧光定量PCR仪(Bio-Rad公司)。

#### 1.2.2 主要试剂

包括:Trizol(上海invitrogen公司), 焦碳酸二乙酯(DEPC)(Sigma公司), AMV逆转录试剂盒(美国Promega公司), 琼脂糖(美国BBI公司), HER2、β-actin引物和探针(上海生工生物工程技术服务有限公司); 热启动荧光PCR核心试剂盒(上海轩昊科技公司); dNTPs、TaqDNA聚合酶、100 bp DNA Marker均购自北京天根生化科技有限公司; 氯仿、乙醇、异丙醇(均为分析纯)购自北京鼎国生物技术有限公司。引物序列如下:HER2(120 bp):F:5'-TCCTGTGTGGACCTGGATGAC-3';R:5'-CCAAAGACCACCCCCAAGA-3';β-actin(207 bp)F:5’-TCCTTCCTGGGCATGGAG-3';R:5'-AGGAGGAGCAATGATCTTGATCTT-3'。TaqMan探针序列如下:HER2:5'(FAM)-AGCAGAATGCCAACCACCGCAGA-(TAMRA)-3', β-actin:5'(FAM)-CCTGTGGCATCCACGAAACTACCTTC-(TAMRA)-3'。

#### 1.2.3 实验方法

*HER2*基因和内标基因*β-actin*在不同的反应管中分别扩增, 均为20 μL PCR反应体系:热启动荧光PCR核心试剂10 μL、上、下游引物(10 μmol/L)各0.5 μL、TaqMan探针(10 μmol/L)3 μL、cDNA 2 μL、双蒸水4 μL。实时定量PCR扩增参数:预变性94 ℃、10 min; 94 ℃变性30 s, 60 ℃延伸1 min, 延伸结束时检测荧光信号, 共40个循环。

为了验证方法的特异性, 将*HER2*与*β-actin*基因的PCR扩增产物于2%琼脂糖凝胶中电泳。同时将*β-actin*和*HER2*基因的引物互换而探针不变进行RT-Q-PCR检测, 观察有无荧光信号响应。为了观察RT-Q-PCR的扩增效率, 取1份cDNA标本分别进行10倍倍比稀释作为*HER2*与*β-actin*基因扩增的模板, 每个基因各5管(5个浓度), 进行RT-Q-PCR并描绘cDNA对数浓度与Ct值的相关曲线, 观察曲线的斜率和相关系数, 通过斜率计算扩增效率, 公式为E=10^-1/k^-1(E为扩增效率, K为标准曲线的斜率)。

### 1.3 统计分析方法

#### 1.3.1 实验数据处理

为了便于对所检测样本进行比较, 在RT-Q-PCR反应的指数期, 首先需设定一定荧光信号的阈值, 一般这个阈值是以PCR反应的前15个循环的荧光信号作为荧光本底信号。如果检测到荧光信号超过阈值被认为是真正的信号, 它可用来定义样本的阈值循环数(threshold cycles, Ct)。每个模板的Ct值与样品中起始模板的拷贝数的对数存在线性反比关系, 起始拷贝数越多, Ct值越小。每个标本重复3次, Ct值取平均值, ΔCt值=*HER2*基因Ct值-*β-actin*基因Ct值, 以β-actin为对照; ΔΔCt值=肺癌组织HER2的ΔCt值-癌旁组织HER2的ΔCt值, 以癌旁组织为对照。以癌旁组织的*HER2*基因表达量为1, 2^-ΔΔCt^值即为肺癌组织相对癌旁组织*HER2*基因表达的倍数, 测定值以2^-ΔΔCt^≥2作为高表达的标准^[7]^。

#### 1.3.2 统计分析方法

所有实验数据使用EpiData 3.0软件建立数据库。运用SPSS 16.0软件进行统计分析, 肺癌组织与癌旁组织中*HER2*基因表达水平的差异分析采用*Wilcoxon*符号秩和检验, *P* < 0.05为差异有统计学意义。

## 2 结果

### 2.1 总RNA的纯度和完整性分析

所提取的总RNA经1%琼脂糖凝胶电泳检测, 可见两条清晰的28S和18S RNA条带, 5S隐约可见(图 1), 且加样孔未见亮带。表明提取的RNA质量完好且无DNA污染。取2 μL提取的RNA稀释500倍, 用紫外分光光度计检测其OD值, *A*_260_/*A*_280_=1.8-2.0, 表明RNA纯度较高, 符合分析的要求。

**1:**
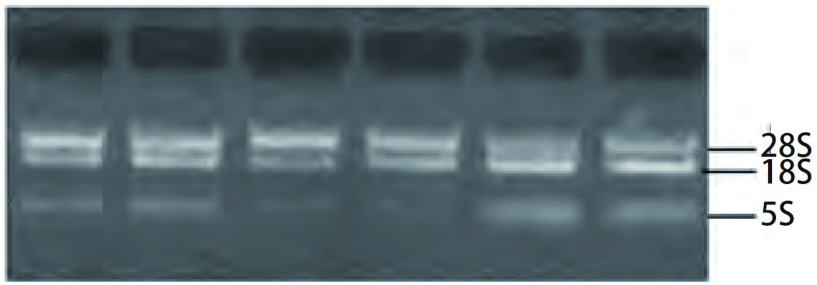
RNA的1%琼脂糖凝胶电泳图 The electrophoretogram of RNA in 1% concentration of agarose gel

### 2.2 RT-Q-PCR扩增产物鉴定

RT-Q-PCR扩增产物经2%琼脂糖凝胶电泳, 在紫外灯下可观察到预期大小与目的片段一致的电泳条带(图 2)。

**2:**
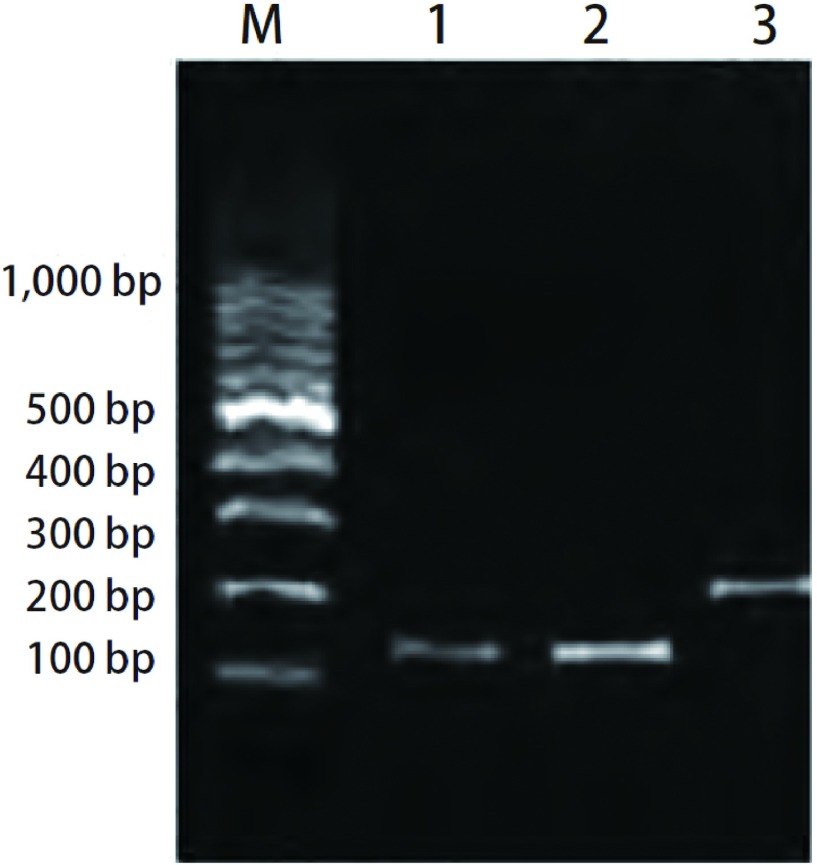
实时定量PCR扩增产物琼脂糖凝胶电泳图。M:分子量标志; 1, 2:*HER2*基因扩增片断; 3:*β-actin*基因扩增片断。 The electrophoretogram of real-time quantitative PCR products in agarose gel.M:DNA marker; 1, 2:Fragments of *HER2* gene amplification; 3:Fragments of *β-actin* gene amplification.

### 2.3 RT-Q-PCR检测*HER2*基因mRNA的动力曲线

将cDNA模板各加入不同的反应管中, 为了检验该方法的重复性, 每个基因同一浓度的cDNA设3个复孔。在icycler iQ荧光定量PCR仪上进行扩增, 结果显示同一基因的扩增动力曲线基本重叠(图 3)。从图中可看出HER2和β-actin扩增的动力曲线都呈S型曲线的形态。

**3:**
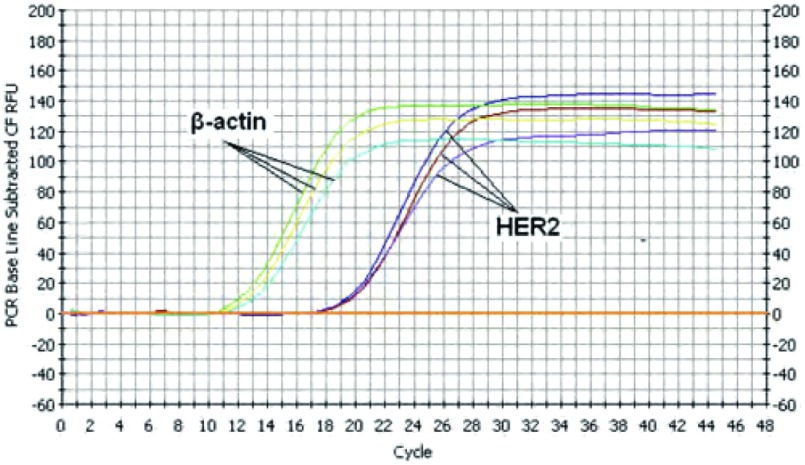
*HER2*和*β-actin*基因实时定量PCR扩增动力曲线图 The dynamical curve of real-time quantitative PCR for *HER2* and *β-actin* genes

### 2.4 RT-Q-PCR特异性测试和扩增效率检测

*HER2*与*β-actin*基因扩增产物电泳结果仅显示一条与目的片段大小相符合的条带(图 2)。将*β-actin*和*HER2*基因的引物互换, 探针不变, 同时进行实时荧光检测, 结果显示, 在45个循环内未检出阳性信号, 说明探针的特异性良好。

分别把10倍倍比稀释的cDNA模板进行*HER2*和*β-actin*基因的实时定量PCR扩增, 各5个浓度, 每个浓度设3个复孔。计算出每个浓度*HER2*和*β-actin*基因的平均Ct值以及ΔCt值, 通过ΔCt值对cDNA的对数浓度值作图, 如果所得直线斜率绝对值接近于0, 说明目的基因和内标基因的扩增效率相同, 就可以通过RT-Q-PCR 2^-ΔΔCt^方法进行相对定量。在图 4A中, 直线斜率是-0.044, 5, 接近0, 说明可以用RT-Q-PCR 2^-ΔΔCt^相对法进行分析数据。如果所有的PCR反应有基本一致的扩增效率, 就可以用比较Ct法的相对定量策略, 而无需每次PCR时做相对定量的标准曲线。分别以*HER2*和*β-actin*基因的Ct值对相应的cDNA对数浓度作图(图 4B), *HER2*和*β-actin*基因对应的相关系数分别为0.999, 9和0.997, 6, 斜率分别为-3.424, 9和-3.405, 2, 扩增效率分别为96%和97%。

**4:**
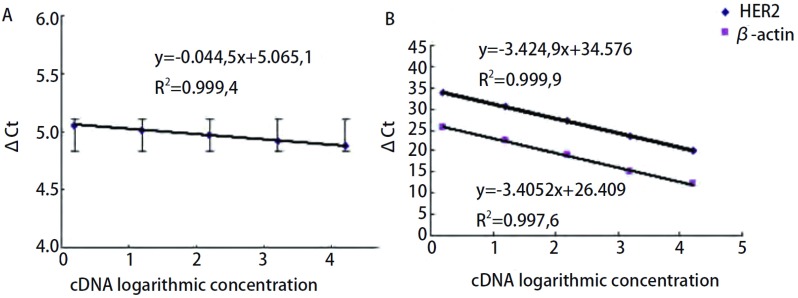
*HER2*和*β-actin*基因的实时定量PCR扩增效率检测。A:*HER2*和*β-actin*基因的△Ct与cDNA对数浓度的关系曲线; B:*HER2*和*β-actin*基因的Ct与cDNA对数浓度的关系曲线。 The amplification efficiency detection of real-time quantitative PCR for *HER2* and *β-actin* genes.A:the curve of the relation between ΔCt and cDNA logarithmic concentration for *HER2* and *β-actin* genes.B:the curves of the relation between Ct and cDNA logarithmic concentration for *HER2* and *β-actin* genes.

### 2.5 *HER2*基因在肺癌组织与癌旁组织表达的差异及在肺癌组织中的过表达率

经检测发现, 212例肺癌组织和其相应的癌旁组织*HER2*基因均有表达, 肺癌组织*HER2*基因的中位数表达水平为4.67(0.21-48.74), 癌旁组织*HER2*基因的中位数表达水平为3.17(0.28-21.26), 肺癌组织高于癌旁组织, 差异具有统计学意义(*P* < 0.01)。在212例NSCLC中, 有72例肺肿瘤组织的*HER2*基因表达水平高于癌旁肺组织2倍或2倍以上, 过表达率为34.0%(72/212)。

## 3 讨论

HER2是EGFR家族中表达相当广泛的受体, 在许多上皮源性的肿瘤细胞中过表达^[8, 9]^。以往肺癌*HER2*基因表达的研究主要基于免疫组化, 使用荧光原位杂交(fluorescence in situ hybridization, FISH)和RT-Q-PCR的较少。FISH相对于其它方法有更高的准确性^[10]^, 但步骤比较繁琐和复杂。而RT-Q-PCR相对于前两者有其独特的优势, 因为RT-Q-PCR可以基于匹配的非肿瘤组织的背景表达水平对每一个样本目的基因的表达水平进行比较精确的测量, 有高通量处理样本的能力和较宽的动力学范围, 能够有效地避免交叉污染和实现操作自动化。事实上, 在评价乳腺癌*HER2*基因表达的状况时, RT-Q-PCR已经被提出作为FISH和IHC的替代方法^[11]^。

RT-Q-PCR分析可以分为相对定量和绝对定量两类。相对定量法更加简单、经济, 而且也更为准确和可靠, 因为存在于反应体系中的干扰因素, 如样本不纯、试剂不同批次等因素的影响都可以通过加入管家基因作为内标准进行校正而去除。采用实时PCR 2^-ΔΔCt^相对定量法比较目的基因在不同样本中的表达差异必须满足一定的条件, 本研究结果显示, 在ΔCt与cDNA对数浓度关系曲线中, 斜率为-0.044, 5, 接近0, 说明目的基因和管家基因的扩增效率基本一致; Ct值对相应的cDNA对数浓度作图, HER2和β-actin对应的相关系数分别为0.999, 9和0.997, 6, 斜率分别为-3.424, 9和-3.405, 2, 扩增效率分别为96%和97%, 表明有良好的线性关系和接近100%的扩增效率。因此, 本研究所采用的RT-Q-PCR 2^-ΔΔCt^相对定量法满足了其运用的基本条件, 表明该方法的应用是可行的, 无需每次测定制作标准曲线, 只需比较不同组织目的基因与内标基因的ΔCt值, 即ΔΔCt值即可。

本实验结果显示, 肺癌组织相对于癌旁组织*HER2*基因的表达水平明显上调(*P* < 0.01)。在212例NSCLC标本中, 72例为过表达(2^-ΔΔCt^≥2), 过表达率为34.0%(72/212), 与Brabender等^[12]^和Pellegrini等^[4]^的报道基本一致, 提示RT-Q-PCR 2^-ΔΔCt^法用于检测NSCLC *HER2*基因的表达是可行的。
